# Management of the congenital solitary kidney: consensus recommendations of the Italian Society of Pediatric Nephrology

**DOI:** 10.1007/s00467-022-05528-y

**Published:** 2022-06-17

**Authors:** Claudio La Scola, Anita Ammenti, Cristina Bertulli, Monica Bodria, Milena Brugnara, Roberta Camilla, Valentina Capone, Luca Casadio, Roberto Chimenz, Maria L. Conte, Ester Conversano, Ciro Corrado, Stefano Guarino, Ilaria Luongo, Martino Marsciani, Pierluigi Marzuillo, Davide Meneghesso, Marco Pennesi, Fabrizio Pugliese, Sara Pusceddu, Elisa Ravaioli, Francesca Taroni, Gianluca Vergine, Licia Peruzzi, Giovanni Montini

**Affiliations:** 1 Pediatric Nephrology and Dialysis, Pediatric Unit, IRCCS Azienda Ospedaliero-Universitaria Di Bologna, Via Massarenti 11, 40138 Bologna, Italy; 2 Pediatric Multi-Specialistic Unit, Poliambulatorio Medi-Saluser, Parma, Italy; 3 Division of Nephrology, Dialysis, Transplantation, and Laboratory On Pathophysiology of Uremia, Istituto G. Gaslini, Genova, Italy; 4 Pediatria C, Ospedale Donna Bambino, Verona, Italy; 5 Pediatric Nephrology Unit, Regina Margherita Department, Azienda Ospedaliero-Universitaria Città Della Salute E Della Scienza, Torino, Italy; 6 Pediatric Nephrology, Dialysis and Transplant Unit. Fondazione Ca’ Granda IRCCS, Policlinico Di Milano, Milano, Italy; 7 Unità Operativa Complessa Di Pediatria E Neonatologia, Ospedale Di Ravenna, AUSL Romagna, Ravenna, Italy; 8 Unità Operativa Di Nefrologia Pediatrica Con Dialisi, Azienda Ospedaliero-Universitaria G. Martino, Messina, Italy; 9 Department of Pediatrics, Infermi Hospital, Rimini, Italy; 10 Institute for Maternal and Child Health—IRCCS Burlo Garofolo, Trieste, Italy; 11 Pediatric Nephrology, “G. Di Cristina” Hospital, Palermo, Italy; 12 Department of Woman, Child and of General and Specialized Surgery, Università Degli Studi Della Campania “Luigi Vanvitelli, Napoli, Italy; 13 Unità Operativa Complessa Di Nefrologia E Dialisi, AORN Santobono – Pausilipon, Napoli, Italy; 14 Unità Operativa Di Pediatria E Terapia Intensiva Neonatale-Pediatrica, Ospedale M Bufalini, Cesena, Italy; 15 Unità Operativa Complessa Di Nefrologia Pediatrica - Dialisi E Trapianto, Dipartimento Di Salute Della Donna E del Bambino, Azienda Ospedaliero-Universitaria Di Padova, Padova, Italy; 16 Pediatric Nephrology Unit, Department of Pediatrics, Marche Polytechnic University, Ancona, Italy; 17 Pediatria AUSL, Imola, Italy; 18 Giuliana and Bernardo Caprotti Chair of Pediatrics, Department of Clinical Sciences and Community Health, University of Milan, Milano, Italy

**Keywords:** Congenital solitary kidney, Congenital anomalies of the kidney and urinary tract, Multicystic dysplastic kidney, Renal agenesis, Renal aplasia

## Abstract

**Background:**

In recent years, several studies have been published on the prognosis of children with congenital solitary kidney (CSK), with controversial results, and a worldwide consensus on management and follow-up is lacking. In this consensus statement, the Italian Society of Pediatric Nephrology summarizes the current knowledge on CSK and presents recommendations for its management, including diagnostic approach, nutritional and lifestyle habits, and follow-up.

**Summary of the recommendations:**

We recommend that any antenatal suspicion/diagnosis of CSK be confirmed by neonatal ultrasound (US), avoiding the routine use of further imaging if no other anomalies of kidney/urinary tract are detected. A CSK without additional abnormalities is expected to undergo compensatory enlargement, which should be assessed by US. We recommend that urinalysis, but not blood tests or genetic analysis, be routinely performed at diagnosis in infants and children showing compensatory enlargement of the CSK. Extrarenal malformations should be searched for, particularly genital tract malformations in females. An excessive protein and salt intake should be avoided, while sport participation should not be restricted. We recommend a lifelong follow-up, which should be tailored on risk stratification, as follows: low risk: CSK with compensatory enlargement, medium risk: CSK without compensatory enlargement and/or additional CAKUT, and high risk: decreased GFR and/or proteinuria, and/or hypertension. We recommend that in children at low-risk periodic US, urinalysis and BP measurement be performed; in those at medium risk, we recommend that serum creatinine also be measured; in high-risk children, the schedule has to be tailored according to kidney function and clinical data.

**Supplementary Information:**

The online version contains supplementary material available at 10.1007/s00467-022-05528-y.

## Introduction and purpose

Congenital solitary kidney (CSK) occurs in approximately 1 in 2000 births and is associated with other congenital anomalies of the kidney and urinary tract (CAKUT) in about one in three cases [1, 2]. Several indications and protocols for follow-up have been issued [3–8] and, most recently, an overview of the clinical aspects and the long-term guidance for children with a congenital solitary functioning kidney has been published [9]. However, a worldwide consensus is still lacking. In this paper, we summarize the current knowledge on the subject and present the recommendations for the diagnosis, evaluation, and follow-up of CSK of the Italian Society of Pediatric Nephrology (SINePE), prepared by the CAKUT Working Group of the Society. Our recommendations are intended for use by all physicians dealing with neonates, infants, and children born with a solitary kidney, in or outside the hospital setting, and by specialists in pediatric and adult nephrology and urology. They can also be used for comparison with other available protocols and overviews.

## Methods

The CAKUT working group of the SINePE, comprising 25 members (pediatric nephrologists and pediatricians), identified the questions and topics concerning CSK to be addressed in the recommendations in an initial working group meeting. After the main topics were approved, members were divided into six sub-groups, each working on one or more questions; four pediatric nephrologists acted as a core leadership group (two as coordinators and writing committee, two as supervisors and revisers). Each sub-group performed a literature search and data extraction, using Excel sheets with specific fields to complete, and wrote the first draft of the answers to the assigned questions, grading evidence according to SORT criteria: strong (grade A), moderate (grade B), and weak (grade C) [10]. After this preliminary work, each draft and the relative recommendations were discussed in a further working group meeting, on the basis of which each group reviewed and completed their draft for the assigned questions. Then the writing committee drafted the whole manuscript, which was revised with the two supervisors and circulated to the whole working group for final approval. Finally, recommendations that were considered to be more controversial were submitted to the members of SINePE and of the CAKUT Working Group of the European Rare Kidney Disease Reference Network (ERKNet) by means of the Delphi method. There were 86 answers (57 from SINePE and 29 from ERKNet members). Recommendations that did not reach a consensus of at least 75% were revised by the writing committee and the supervisors, considering the suggestions made by the Delphi participants, and approved by the Working Group.

### Literature search

The PICO (Patient or Population covered, Intervention, Comparator, Outcome) [11] framework was used to develop our search strategies and to formulate the questions for the consensus recommendations. Population was children with CSK; Interventions were 1 — diagnostic approach; 2 — evaluation of kidney function, signs of kidney damage, and blood pressure (BP); 3 — use of kidney protective medications, nutritional and lifestyle habits, and sports participation. Comparators were either the adoption or non-adoption of interventions for diagnosis, medical treatment, and follow-up. Outcomes were accuracy in the diagnosis of CSK, kidney function and BP, and quality of life. A PubMed search was performed from January 2000 to January 2021 using the following key words: congenital solitary kidney, unilateral renal agenesis, multicystic dysplastic kidney (MCDK), solitary kidney, renal dysplasia, and the following limits: humans, child, and English. The search retrieved 995 articles; from this list, we selected cohort studies, metanalyses, and systematic reviews related to our above-mentioned interventions; no randomized clinical trials were available. One hundred and forty-nine articles were examined. Furthermore, a manual search was carried out, mainly using relevant references from the analyzed articles, which identified a further 21 articles, bringing the total to 170. From these, a final evaluation identified the 72 articles which were pertinent to the analyzed interventions and topics, and which provided appropriate data, and these are referenced in our consensus recommendations (Supplementary).

### Statistical analysis

All statistical analyses were performed by the open-source software R (R Core Team (2021) R: A language and environment for statistical computing. R Foundation for Statistical Computing, Vienna, Austria. URL https://www.R-project.org/).

In particular, weighted means, standard deviations, medians, and interquartile ranges (IQRs) were computed using the R package Hmisc (Frank E Harrell Jr, with contributions from Charles Dupont and many others. (2020) Hmisc: Harrell Miscellaneous. R package version 4.4–0. https://CRAN.R-project.org/package=Hmisc).

Weighted distributions were computed using the rates of patients in each study to the total number of patients as weights.

## Definition and classification

Congenital solitary kidney is the anatomical or functional absence of one kidney from birth. The former is due to a complete failure of embryonic kidney formation (agenesis), the latter to extreme forms of dysplasia causing absence of function: aplasia and MCDK. This embryologic differentiation is usually possible when a CSK is suspected in the second trimester of pregnancy, mainly on the ultrasound (US) scan between the 18th and the 22nd weeks of gestation [12]. However, a dysplastic kidney may regress during fetal life; this is well known for MCDK, which can involute completely as early as the 29th week of pregnancy [8]. Therefore, the involuted dysplastic kidney becomes undetectable at US, and in the presence of an empty kidney fossa, it becomes difficult to ascertain whether it is due to agenesis, aplasia, or an involuted MCDK. In these cases, the exact classification is not possible, and the embryologic origin of the CSK has to be left undefined [7].

### Statements/recommendations:

We recommend that CSK be classified as follows:

- Agenesis: absence of one kidney suspected on US scan between the 18th and 22nd weeks of gestation and confirmed postnatally.
- Aplasia: a rudimentary kidney suspected on US scan between the 18th and 22nd weeks of gestation, with relative function < 5% at 99mTc-dimercaptosuccinic acid scintigraphy (DMSA).
- MCDK: multiple non-communicating cysts of various sizes within a lobulated renal contour, pelvis and parenchyma not being visible on US.
- Undefined CSK: detection of an empty kidney fossa in the third trimester of pregnancy or after birth, with the differential diagnosis of agenesis, aplasia, or an involuted MCDK remaining uncertain (grade C).

## What to do when a CSK is suspected/diagnosed by ultrasound prenatally?

The suspicion of a CSK can be raised by antenatal US screening, starting with the morphology scan at around 20 weeks of gestation; it can be substantiated by the detection of a compensatory enlargement of the CSK, which occurs in up to 88% of fetuses and can be demonstrated as early as the 20th week of gestation [13]. However, the anatomical absence of one kidney may not be detected in utero for a number of reasons: the adrenal gland can fill the kidney fossa and may be mistaken for the kidney; late in the second or in the third trimester, the retroperitoneal colon can suggest the presence of a kidney; an initially visible dysplastic kidney may regress to the point of becoming undetectable by US (approximately 5% of MCDKs) [8, 12]. Data from European collaborative registries on 709,030 births [14] showed that unilateral kidney agenesis (UKA) was correctly detected by prenatal US in 62% of cases, if isolated, and in 80% of cases when associated with other malformations; in an Italian cohort, a CSK was correctly identified prenatally in 62% of patients [7]. Antenatal US has other caveats: the kidney fossa may be empty because of kidney ectopia, which may not be identified, and the prenatal distinction between MCDK and severe hydronephrosis may be difficult: in a group of patients with MCDK, 30% (15/50) were erroneously diagnosed with hydronephrosis [15].

A prenatally identified CSK is diagnosed as an isolated anomaly in approximately two thirds of cases, but is associated with multiple malformations (syndromic or non-syndromic), in about a third of patients [14]. For this reason, if a CSK is described, it is important that a specialized US scan of the whole fetus be performed [12]. If the CSK is isolated, we believe that standard obstetric follow-up is warranted. In the presence of additional fetal structural malformations, the risk of genetic abnormalities is increased, and may surpass 50% for some associations [16]. In these cases, chorionic villus sampling or amniocentesis with chromosomal microarray/karyotyping should be offered [16].

### Statements/recommendations:

1. We recommend that a specialized US examination of the whole fetus be performed when a CSK is diagnosed prenatally (grade B).
2. We recommend that in fetuses with a diagnosis of CSK as an isolated malformation no diagnostic procedure other than standard obstetric follow-up be performed (grade C).
3. We recommend that in fetuses with a diagnosis of CSK associated with extrarenal malformations, chorionic villus sampling or amniocentesis with chromosomal microarray/karyotyping should be offered (grade B).
4. We recommend that any antenatal suspicion/diagnosis of CSK or detection of abnormal kidney or urinary tract morphology be confirmed by neonatal US (grade B).

## How should a CSK be confirmed postnatally?

The prenatal suspicion/detection of a CSK has to be substantiated by a neonatal US scan.

The postnatal US report should always describe kidney length, echogenicity and parenchymal thickness, the features of the calyces, and the antero-posterior diameter of the renal pelvis where it exits the parenchyma. Other important characteristics are the maximal ureteric diameter, if visible, bladder wall thickness and, if possible, pre-void and post-void bladder volume.

Although scintigraphy is the gold standard for confirming the functional absence of a kidney, advances in US technology mean that nuclear scans are not necessary in most cases, thus avoiding radiation exposure. In a study involving 128 CSK patients (agenesis 75, MCDK 53) confirmed by nuclear scan, neonatal US detected CSK in the vast majority of patients; however, it missed 8.6% of cases, which were all MCDK [17]. In that study, neonatal US had a specificity of 100% and a sensitivity of 92.1%: sensitivity was 100% for agenesis and aplasia and 82.8% for MCDK. The positive and negative predictive values were 100% and 99.9%, respectively. In another study, US correctly diagnosed a CSK, as confirmed by nuclear scanning, in 24/25 infants; in 1 patient, US suggested a pelvic kidney but repeat US was negative, as was the DMSA scan [18]. Whittam et al. verified the absence of function in 84/84 MCDK patients with a nuclear scan, previously diagnosed by postnatal US [15]. The possibility that an ectopic kidney may be missed by US is not substantiated by the above-mentioned literature; moreover, the presence of a rudimentary ectopic kidney would be irrelevant for management and follow-up. For these reasons, we believe that a postnatal US scan performed by an experienced pediatric radiologist is, in most cases, sufficient for the definitive diagnosis of a CSK. In questionable cases and/or if US cannot distinguish between an MCDK and severe hydronephrosis, a more extensive imaging work-up (mercaptoacetyltriglycine scintigraphy or magnetic resonance urography) has been suggested [19].

### Statements/recommendations:

1. For the definitive diagnosis of CSK, a neonatal US performed by an experienced pediatric radiologist is sufficient in most cases (grade B).
2. We do not recommend the routine use of scintigraphy to confirm the anatomical or functional absence of a kidney (grade B).
3. Further imaging is recommended in the event of the uncertain diagnosis of a rudimentary kidney (DMSA) or of a doubtful differential diagnosis between MCDK and severe hydronephrosis (mercaptoacetyltriglycine scintigraphy or magnetic resonance urography) (grade C).

## What further imaging is required when a CSK is confirmed?

Having diagnosed the anatomical or functional absence of one kidney, the next step is to exclude an anomaly of the contralateral urinary tract, as about one in three patients has additional CAKUT [1, 2]. While US can raise the suspicion of many anomalies of the urinary tract (i.e., pelvi-ureteric junction obstruction, megaureter, duplex system), it has a low predictive value for the presence of vesicoureteral reflux (VUR) [20].Thus, three main questions have to be addressed:

- a. Is the CSK normal at US?
- b. Should imaging to detect VUR be performed routinely?
- c. When should further imaging for associated uropathies be performed?

### a) Is the CSK a normal kidney?

**Fig. 1:**
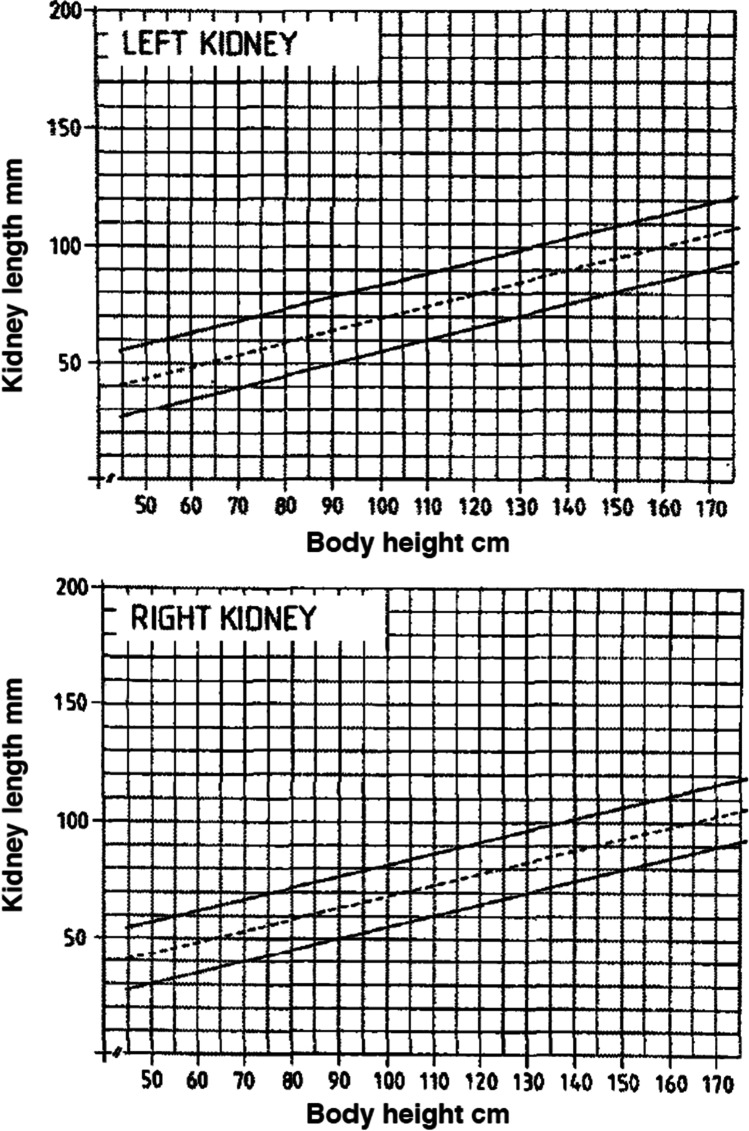
Sonographical growth charts for kidney length related to height (5th, 50th, and 95th percentiles). Reproduced from Dinkel et al. [25], used with permission

A CSK without additional abnormalities is expected to undergo compensatory growth. Whether this enlargement is due to the hypertrophy of existing nephrons or to an increase in the number of nephrons formed in utero, leading to hyperplasia, is still debated [7, 21]. Compensatory enlargement can start during gestation, and can be detected by current US techniques sometimes as early as the 20th week [13]. However, compensatory growth is often established in the first year of life, or beyond in some cases [22, 23]. Once a CSK has undergone compensatory enlargement, its length/size does not subsequently regress [22]. To evaluate if compensatory growth of the CSK is taking place, US measurements of kidney length can be used, comparing them with normative data. At present, nomograms constructed from US measurements to assess kidney length in children with two kidneys [24–26] are used. Compensatory enlargement of the CSK in children is defined either in relation to age (length ≥ 2 or >2.5 SD) [23, 27–33], or in relation to height (≥ 95th percentile) [7, 17, 22, 34–37], while in adults, the expected value is ≥ 120 mm [38, 39]. As there is a wide variability of physical growth with age, we believe that relating kidney length to height rather than to age is more appropriate. For that purpose, we suggest using the nomograms published by Dinkel et al. (Figure 1) [25]. Alternatively, the web-based tool published by Chen et al., which requires multiple demographic variables, could be used (available at: https://www.prevmed.sunysb.edu/jjc/MrNomogram/default2.aspx) [26]. However, it should be remembered that in conditions which distort normal kidney anatomy, such as hydronephrosis, a duplex collecting system, or an ectopic kidney, kidney length may not accurately reflect the compensatory enlargement of the parenchyma: in these cases, the available nomograms of parenchymal area for CSK should be considered [27]. Furthermore, it is important to bear in mind that the rate of kidney growth is most rapid during the first 2 years of life and that it slows down between 2 and 5 years, after which kidney length increases by only 2–3 mm per year throughout adolescence [40].

We believe that a CSK which does not fulfill the parameters outlined in Box 1 should be considered abnormal.

Box 1 Opinion-based definition of a normal ultrasound for congenital solitary kidney.

**Table:** 

US parameter	Needed description	Normal findings	Timing
Kidney length	Bipolar diameter in millimeters	> 50th percentile ≥ 95th percentile	Until 2 years of life After 2 years of life
Parenchyma	Thickness Echogenicity Cortico-medullary differentiation Cysts	Normal Normal Normal Absent	Any time
Renal pelvis	Antero-posterior diameter in millimeters	≤ 10 mm	> 48 h of life
Calices	Dilatation	Absent	Any time
Ureter	Dilatation	Absent	Any time
Bladder	Wall thickness Ureterocele	Normal Absent	Any time

#### Statements/recommendations:

1. A CSK without additional abnormalities is expected to undergo compensatory growth (grade B).
2. We recommend measuring the US length of a CSK at diagnosis and at each US during follow-up (grade C).
3. We recommend that the length of the CSK be evaluated with nomograms, relating kidney length to body height (grade C).
4. If the kidney shows abnormalities in morphology or position (i.e., it is hydronephrotic or ectopic), length measurements may not reflect parenchymal enlargement; in these cases, measuring the parenchymal area should be considered (grade C).
5. We recommend that the term compensatory enlargement, rather than compensatory hypertrophy, be used as the exact mechanism leading to the increased growth of a CSK is still unknown (grade C).
6. We recommend waiting until 2 years of age to establish the absence of compensatory enlargement (grade C).

### b) Should imaging to detect VUR be performed routinely?

Among the urologic abnormalities associated with CSK, vesicoureteral reflux is the most common [1, 2, 7, 31, 41]. Two systematic reviews involving over 5600 patients with MCDK or UKA, 2874 having been examined for the presence of VUR, showed an overall rate of about one in five patients [1, 2]. However, severe VUR (i.e., grades III–V according to the International Reflux Study classification) appears to be infrequent, having been documented in 9% of children with CSK overall in recent series [1, 7, 31, 41–43] (Table 1).

**Table 1:** Reported prevalence of associated uropathies in children with CSK

Author, year	Number of pts	CSK type (%)	Associated CAKUT, %	Total VUR, %	VUR grades III–V, %	UPJO, %	VUJO, %
**Schreuder M. (2009) **[1]*	3557	MCDK (100)	31.3 (of 2415 pts)	15 (of 2104 pts)	8	4.8 (of 2159 pts)	NR
**Westland R. (2013) **[2]*	1093	UKA (100)	32	24 (of 770 pts)	NR	6 (of 615 pts)	7 (of 605 pts)
**La Scola C. (2016) **[7]	146	MCDK (38), UKA (29), UKAP (16), Undefined (18)	21	11.5	10	2	3
**Ross I. (2015) **[42]	138	MCDK (63), UKA (37)	NR	36	17	NR	NR
**Marzuillo P. (2017)**[31]	322	MCDK (48), UKA (52)	14.6	9.3	5.6	0.3	4
**Brown C. (2019) **[43]	165	MCDK (100)	33	17 (of 77 pts)	NR	NR	NR
**Blachman-Braun R. (2020) **[41]	156	MCDK (100)	NR	16	6	NR	NR

^*^Previous works not analyzed as present in the two metanalyses

*Legend*: *Pts* patients, *CSK* congenital solitary kidney, *CAKUT* congenital anomalies of kidney and urinary tract, *VUR* vesicoureteral reflux, *UPJO* ureteropelvic junction obstruction, *VUJO* vesicoureteral junction obstruction, *MCDK* multicystic dysplastic kidney, *UKA* unilateral kidney agenesis, *UKAP* unilateral kidney aplasia, *NR* not reported

Ultrasound has a low predictive value for the presence of VUR as it often yields normal results in children with low grade and even in some with high grade VUR [20, 44]. Therefore, further imaging procedures are needed to detect VUR, fluoroscopic contrast voiding cystourethrography (VCUG) being the standard. However, from a clinical point of view, low grade VUR is increasingly recognized as negligible, in terms of both the rate of UTI and the risk of kidney scarring, while the role severe VUR plays in clinical outcome is still debated, as conclusive evidence is lacking [45]. Multiple studies have demonstrated a low incidence of clinically significant VUR in children with MCDK and a normal contralateral kidney and bladder on US [41]; furthermore, Brown et al. found that knowledge of VUR in 77 children with MCDK screened by VCUG did not change patient management [43]. Accordingly, we believe that routine screening for VUR is not necessary in the presence of a normal CSK on US (Box 1). In this context, in our opinion, an isolated dilatation of the renal pelvis ≤ 10 mm does not represent an indication for VCUG. On the other hand, it has been shown that in children with an abnormal CSK on US, the probability of a high grade VUR is elevated [41, 46]. In the risk analysis performed by Blachman-Braun et al., a significant association (odds ratio = 7.73; 95%CI: 1.43–41.81; *p* = 0.018) between abnormal CSK on US (defined as abnormal contralateral kidney, presence of hydronephrosis, duplex configuration, ureterocele, hydroureter, or uroepithelial thickening) and severe VUR was demonstrated in 156 MCDK patients [41]. For this reason, we believe that VCUG should be performed when US abnormalities of the CSK or urinary tract are reported (see Box 1).

#### Statements/recommendations:

1. In children with a normal CSK and urinary tract on US (Box 1), routine imaging to rule out the presence of VUR is not recommended (grade B).
2. We believe that VCUG should be performed when abnormalities of the CSK or urinary tract are reported on US (see Box 1) (Grade C).

### c) When should further imaging for associated uropathies be performed?

In general, if a urinary tract anomaly associated with CSK is detected on US, further imaging should be performed as recommended in children with two kidneys, particularly after a febrile UTI [44] and if obstruction is suspected. Special attention should be paid to obstructive uropathies of the CSK, which may be severe enough to cause acute kidney failure [47].

#### Statements/recommendations:

1. If a urinary tract anomaly associated with CSK is detected on US, further imaging should be performed, as indicated in children with two kidneys (grade C).
2. We recommend that special attention be paid to the diagnosis of an obstructive uropathy which, if severe, may cause acute kidney failure in a solitary kidney (grade C).

## Are laboratory tests necessary at diagnosis?

To date, several opinion-based recommendations on the type and timing of laboratory tests in children with CSK have been published [5–7, 9]. In each of these, laboratory testing (in particular, albuminuria or proteinuria, and serum creatinine) is foreseen, with different scheduling based on the presence or absence of an ipsilateral CAKUT. La Scola et al. first introduced the concept of timing based also on kidney length/size and proposed less intensive laboratory assessments for children with an adequate kidney length/size [7]. Poggiali et al. used a prediction model, including neonatal plasma creatinine, kidney length, and history of recurrent UTIs, which enabled the identification of a subgroup of patients with an increased risk of kidney damage, hypertension and/or reduced glomerular filtration rate (GFR) over time [48]. In a more recent paper on the clinical management of children with CSK, it was reported that none of the 46 children with a kidney size of > 2 SDs above the mean for age (for an individual with two kidneys) had a reduced kidney function within the first year of life. Therefore, the authors suggested that in the absence of other clinical indications, it seems reasonable to refrain from an initial creatinine measurement in CSK patients with compensatory hypertrophy [9]. It must also be underlined that the above-mentioned opinion-based recommendations were derived from studies carried out in specialized units, where patients are likely to be the most severely affected, and so selection bias may have played a part. We recommend performing urinalysis, to rule out proteinuria, but not routine blood tests, at diagnosis in infants and children with normal US measurements for CSK, as delineated in Box 1; if routine urinalysis shows proteinuria, a quantitative assessment should be performed [49]. In children with abnormal US measurements of the CSK or in the presence of a clinical indication, plasma creatinine and quantitative proteinuria have to be evaluated.

### Statements/recommendations:

1. We recommend performing urinalysis, to rule out proteinuria, but not routine blood tests, at diagnosis in infants and children with normal US measurements for CSK, as delineated in Box 1 (grade C).
2. In all infants and children with an abnormal CSK on US, plasma creatinine and quantitative proteinuria have to be evaluated at diagnosis (grade C).

## When and how should extra-renal malformations be searched for?

Extra-renal malformations can be associated with CSK, with a prevalence described between 6 and 31% in hospital-based series [1, 2, 7, 31, 34, 50] (Table 2). In the aforementioned systematic reviews, extra-renal malformations were described in approximately 15% of 1340 subjects with MCDK [1] and in 31% of 709 subjects with UKA [2]. These malformations occurred as part of specific multi-organ syndromes in up to 10% of patients [7, 31, 34]. The most frequently described extra-renal malformations involve the heart, the gastrointestinal tract, and the musculoskeletal and genital apparatuses. Therefore, a careful clinical examination must always be performed in subjects with CSK, both at diagnosis and during follow-up, in order to detect signs of extra-renal malformations needing further work-up. If extra-renal malformations are detected, the possible presence of a syndrome (Table 3) and the need for genetic counseling/analysis have to be considered [3]. An association which calls for special attention is that between CSK and genital malformations [2, 51–53]. This association, although occasionally present in males (seminal vesicle hypoplasia and absence of the vas deferens), is more common in females: female tract malformations were described in 11% of 502 patients with UKA in the metanalysis performed by Westland [2]. They may include uterine and vaginal agenesis, uterine duplicity (didelphys, bicornuate or septate uterus), obstructed or blind hemivagina, monolateral ovarian agenesis, and Gartner duct pseudocyst. These malformations are frequently linked to syndromes (Table 3). As prenatal US is not a reliable method for genital malformation screening, and as some of these conditions can be asymptomatic during childhood, their detection is often delayed until after menarche, when serious complications caused by obstructive anomalies can arise [51]. For this reason, an abdominopelvic US must be performed in all girls with a CSK between thelarche and menarche, to exclude genital abnormalities [51].

**Table 2:** Reported prevalence of extrarenal malformations in children with congenital solitary kidney

	**Number of pts**	**Genital**	**Cardiac**	**Musculo-skeletal**	**Inguinal hernia**	**Haemato-poietic**	**Eye**	**Nervous**	**Endocrine**	**Gastro-intestinal**	**Respiratory**	**Syndromes**	Total***
**Schreuder M. (2009) **[1]*	1340	NR	NR	NR	NR	NR	NR	NR	NR	NR	NR	NR	**14.9%**
**Mansoor O. (2011) **[34]	101	-	3%	-	-	-	-	-	-	-	-	3%	**6%**
**Westland R. (2013) **[2]*	709	11%**	14%	13%	NR	NR	NR	NR	NR	16%	NR	NR	**31%**
**La Scola C. (2016) **[7]	146	9%	7.5%	7.5%	2%	1.4%	-	6.1%	5.5%	3.4%	1.4%	10.3%	**26%**
**Groen in’t Wood S. (2016) **[50]	49	NR	NR	NR	NR	NR	NR	NR	NR	NR	NR	NR	**12.2%**
**Marzuillo P. (2017) **[31]	322	3.4%	3.1%	0.3%	1.2%	0.6%	0.6%	-	-	-	-	1.2%	**10.5%**

^*^Previous works not analyzed because present in the two metanalyses; ** of 502 females; ***some patients could have more than one malformation

Legend: NR not reported, pts patients, — zero

**Table 3:** Most reported syndromes in association with congenital solitary kidney

Syndrome	Extrarenal manifestations	Genes	Possible inheritance
Branchio-oto-renal	Sensorineural hearing loss, preauricular pits, branchial cysts, and microtia	*EYA1, SIX1, SIX5*	Autosomal dominant
DiGeorge	Congenital heart disease, hypocalcaemia, immunodeficiency, and neurocognitive disorders	22q11 deletion	Autosomal dominant
Fraser	Cryptophthalmos, cutaneous syndactyly, occasionally malformations of the larynx, ambiguous genitalia, and mental retardation	*FRAS1, FREM2*	Autosomal recessive
Herlyn-Werner-Wunderlich or OHVIRA (obstructed hemivagina and ipsilateral renal agenesis)	Obstructed hemivagina and uterus didelphys	Unknown	Autosomal dominant
Kallmann 1	Micropenis, bilateral cryptorchidism, and anosmia	*KAL1*	X-linked
Klinefelter	Small, firm testis, gynaecomastia, azoospermia, and hypergonadotropic hypogonadism	47, XXY	Sporadic
MURCS (Mayer-Rokitansky-Kuster–Hauser type 2)	Müllerian duct aplasia-hypoplasia and cervicothoracic somite dysplasia	Unknown	Autosomal dominant
Renal coloboma	Retinal and optic nerve coloboma	*PAX2*	Autosomal dominant
Renal cysts and diabetes	Maturity-onset diabetes of the young type 5, hyperuricaemia, hypomagnesemia, and uterine malformations	*HNF1B*	Autosomal dominant
Townes–Brocks	Thumb anomalies, imperforate anus, and sensorineural hearing loss	*SALL1*	Autosomal dominant
VACTERL association	Vertebral anomalies, anorectal malformations, cardiovascular disease, tracheoesophageal fistula, esophageal atresia, and limb defects	*TRAP1*	Autosomal recessive
Williams–Beuren	Developmental delay, cardiovascular anomalies, mental retardation, and facial dysmorphology	7q11.23 deletion	Autosomal dominant

### Statements/recommendations:

1. We recommend that a careful clinical examination be performed at diagnosis and during follow-up, to detect signs of extra-renal malformations needing further work-up (grade C).
2. In all girls with a CSK, we recommend screening for genital malformations by means of an abdominopelvic US, between thelarche and menarche (grade B).
3. If extra-renal malformations are detected, the possible presence of a syndrome and the need for genetic analysis and/or counseling have to be considered (grade A).

## Is it necessary to perform genetic analysis in non-syndromic forms of CSK?

Genetic studies specifically focused on CSK are rare, as this malformation is usually studied in the context of CAKUT. At present, from a genetic point of view, the cause remains speculative in approximately 80% of CAKUT cases, with several studies supporting a multifactorial pathogenesis [54]. In a recent study on 86 patients with UKA, 9 subjects (10.5%) showed pathogenic mutations in seven different genes [55]. Ishiwa et al., in a study on 66 patients with severe CAKUT (i.e., associated to extreme forms of dysplasia with bilateral kidney lesions, extrarenal complications, or a family history of kidney disease), detected mutations in 10/33 subjects (30%) in whom MCDK or kidney aplasia were associated with a severe malformation of the contralateral CSK [56]. In this series, the detection rate of genetic anomalies was significantly higher in subjects with bilateral kidney lesions than in those with a syndromic CAKUT or a family history of renal malformations. The most frequent genetic anomalies concerned *HNF1Ɓ* and *PAX2* [56]. Van der Ven et al. performed whole-exome sequencing in 232 families in which 319 subjects were affected by CAKUT. In 29/232 families, a mutation in known genes for CAKUT was detected, and in 10/29, the corresponding clinical phenotype showed the presence of UKA or MCDK [57].

Thus, at present, the complex underlying genetic mechanisms of CAKUT and CSK do not allow for the establishment of a common diagnostic approach for all patients, and in each case, the most indicated test and the diagnostic yield should be considered [58], taking into account that in subjects with bilateral kidney lesions, the detection rate of genetic anomalies is higher than in isolated CSK and that a search for genetic anomalies seems reasonable in families with recurrent cases of CAKUT.

### Statements/recommendations:

1. We do not recommend genetic counseling/analysis in children with an isolated and sporadic CSK (grade C).
2. We suggest that genetic counseling be offered to children with a CSK and an ipsilateral CAKUT and/or a family history of CAKUT (grade C).

## Risk of decreasing glomerular filtration rate and of kidney damage (proteinuria and hypertension) over time

In spite of the high variability of nephron numbers in the general population [59], nephron number is likely to be lower in subjects with CSK than in those with two kidneys [60]. When there is a reduced number of functioning nephrons, compensatory physiological and biochemical adaptations occur. In animals with extensive kidney ablation, these adaptations produce glomerular overload and hyperfiltration, resulting in proteinuria, glomerulosclerosis, higher rates of hypertension, and decreasing GFR [61]. In humans, an extensive debate on the prognosis of CSK exists, and some risk factors have been demonstrated to have a negative impact on outcome. Therefore, from a clinical perspective, the challenge is to identify patients at increased risk of kidney damage and GFR reduction, in order to tailor their follow-up accordingly.

Three main questions have to be addressed:

a What is the risk of decreasing GFR over time?
b What is the risk of developing proteinuria?
c What is the risk of developing hypertension?

### a) Is a child with CSK at risk of decreasing glomerular filtration rate?

Studies analyzing kidney function over time in patients with CSK have described a variable prevalence of reduced function both in children [4, 7, 17, 22, 23, 30, 31, 33, 34, 48, 62–66] and in adults [38, 39, 67–69] (Table 4). This high variability was influenced by different inclusion criteria, follow-up periods, and outcome measures: in particular, in some studies, the outcome was GFR < 90 ml/min/1.73 m^2^, in others < 60 ml/min/1.73 m^2^. In addition, many studies were performed in referral hospitals, possibly selecting patients with more serious conditions. In Table 5, we report the calculated weighted means, standard deviations, weighted medians, and IQRs for the outcome GFR reduction, separately for children and adults. Moreover, a stratified analysis for pediatric cohorts according to the GFR threshold (< 90 ml/min/1.73 m^2^ in 9 series, < 60 ml/min/1.73 m^2^ in 4 series) is shown (Table 5). Among the studies with the highest percentage of GFR < 90 ml/min/1.73 m^2^ were those conducted by Hayes [30] and Siomou [66]. Hayes described a measured glomerular filtration rate < 90 in 43% of children; however, 73% had a value between 80 and 89 ml/min/1.73 m^2^. Siomou found an estimated glomerular filtration rate (eGFR) < 90 ml/min/1.73 m^2^ in 42% of children, but the great majority of them (81%) had a value > 80 ml/min/1.73 m^2^. Thus, in these two series, GFR reduction was very mild in most patients.

**Table 4:** Reported adverse outcomes in cohorts of children and adults with congenital solitary kidney

Author, year	Age	*N*	Compensatory enlargement*	Ipsilateral CAKUT	Kidney injury		GFR reduction		Proteinuria/albuminuria		Hypertension	
					**Definition**	**Outcome *N* (%)**	**Evaluated as**	**Outcome *N* (%)**	**Evaluated as**	**Outcome *N* (%)**	**Definition**	**Outcome *N* (%)**
Weinstein A. (2008) [22]	C	80	80%	NR			eGFR < 90 ml/min/1.73 m^2^	0/23	Dipstick > + 1	0/20	> 95th pct	0/31 (0)
Vu K. H. (2008) [4]	C	56	"normal kidneys"	None			eGFR < 90 ml/min/1.73 m^2^	6/56 (10.7)	ACR > 0.25 mg/mg	4/52 (7.7)	> 95th pct	3/56 (5.3)
Abou Jaoudé P. (2011) [62]	C	44	"normal kidneys"	None			mGFR < 90 ml/min/1.73 m^2^	5/44 (11.4%)	UAlb/UCr > 2 mg/mmol	8/44 (18)	> 95th pct	1/44 (2.2)
Mansoor O. (2011)[34]	C	121	74%	25% (of 101 patients)			eGFR < 90 ml/min/1.73 m^2^	8/82 (9.7)	UPr/UCr ≥ 0.2 mg/mg	14/82 (17)	> 95th pct	6/86 (7)
Hayes W. N. (2012) [30]	C	323	97%	14%			mGFR < 90 ml/min/1.73 m^2^	33/76 (43)			NR	0/94 (0)
Stefanowicz J. (2012) [63]	C	17	NR	None			eGFR < 90 ml/min/1.73 m^2^	0/17	ACR > 30 mg/g	4/17 (24)	> 95th pct	1/17 (6)
Westland R. (2013) [64]**	C	223	NR	26%	Hy and/or Proteinuria and/or eGFR < 60 ml/min/1.73 m^2^ and/or RPM	68/223 (31)	eGFR < 60 ml/min/1.73 m^2^	9/223 (4)	UPr/UCr > 0.2 mg/mg (> 0.5 if < 2 yrs) or ACR > 30 mg/g	29/223 (13)	> 95th pct	49/223 (22)
Kolvek G. (2014) [65]	C	27	NR	29%	Hy and/or Proteinuria and/or eGFR < 60 ml/min/1.73 m^2^, and/or RPM	9/27 (33.3)	eGFR < 60 ml/min/1.73 m^2^	3/27 (11)	≥ 300 mg/24 h	2/27 (7.4)	> 95th pct	5/27 (18)
Siomou E. (2014) [66]	C	38	100%	0			eGFR < 90 ml/min/1.73 m^2^	17/38 (44.7)			> 95th pct	4/38 (11)
La Scola C. (2016) [7]	C	146	65%	21%			eGFR < 90 ml/min/1.73 m^2^	18/146 (12)	UPr/UC > 0.2 mg/mg (> 0.5 if < 2 yrs)	5/134 (4)	> 95th pct	6/121 (5)
Marzuillo P. (2017) [31]***	C	322	74%	15%	Hy or Proteinuria or eGFR < 90 ml/min/1.73 m^2^	12/306 (3.9)	eGFR < 90 ml/min/1.73 m^2^	4/306 (1.3)	UPr/UC > 0.2 mg/mg (> 0.5 if < 2 yrs)	11/306 (3.6)	> 95th pct	2/306 (0.6)
Urisarri A. (2018) [17]	C	128	64%	35%			eGFR < 60 ml/min/1.73 m^2^	3/128 (2.3)	ACR 30 mg/g	4/128 (3)	> 95th pct	6/128 (4.6)
Poggiali I. (2019) [48]	C	162	NR	26%	Hy, Proteinuria and eGFR < 60 ml/min/1.73 m^2^	18/162 (11.1)	eGFR < 60 ml/min/1.73 m^2^	9/162 (5.6)	UPr/UCr > 0.2 mg/mg or > 150 mg/day	10/162 (6.2)	> 95th pct	11/162 (6.8)
Sanna Cherchi S. (2009) [68]	A	111	NR	NR			eGFR < 15 ml/min/1.73 m^2^	23/111 (21)	> 1 g/day	5/111 (4.5)	NR	3/111 (2.7)
Argueso L. R. (1992) [67]	A	157	100%	NR			mGFR < 90 ml/min/1.73 m^2^	4/32 (13)	> 150 mg	7/37 (19)	> 160/95 mmHg and/or T	22/47 (47)
Wang Y. (2010) [38]	A	65	NR	NR			mGFR < 60 ml/min/1.73 m^2^	25/65 (38)	ACR > 10 mg/mmol or RPM	31/65 (48)	> 140/90 mmHg and/or T	24/65 (36)
Basturk T. (2015) [39]	A	31	NR	NR	sCr > 1.4 mg/dl in men or > 1.3 mg/dl in women or eGFR < 60 ml/min/1.73 m^2^ or Proteinuria ≥ 300 mg/day	17/31 (55)	mGFR < 60 ml/min/1.73 m^2^	14/31 (45)	UPr ≥ 300 mg/day	12/31 (39)	NR	21/31 (67)
Xu Q. (2019) [69]	A	118	NR	NR	Hy, Proteinuria and eGFR < 60 ml/min/1.73 m^2^	62/118 (53)	eGFR < 60 ml/min/1.73 m^2^	30/118 (25.4)	> 150 mg/day	49/114 (43)	≥ 140/90 mmHg	38/118 (32.2)

^*^Kidney length ≥ 2SD for age or ≥ 95th percentile for height; **includes  also cohorts described in references 29 and 33; ***includes also patients described in reference 23

*Legend*: *CAKUT* congenital anomalies of kidney and urinary tract, *C* children, *A* adults, *Hy* hypertension, *eGFR* estimated glomerular filtration rate, *mGFR* measured glomerular filtration rate, *UAlb* urine albumin, *UPr* urine protein, *UCr* urine creatinine, *ACR* albumin creatinine ratio, *NR* not reported, *RPM* renoprotective medication, *T* therapy

**Table 5:** Weighted distribution of outcomes calculated from the cohorts reported in Tables 4 and 7

**Outcome**	**Population**	**N. of cohorts**	**Mean** ± **sd**	**Median (IQR)**
**GFR reduction**	Pediatric cohorts [4, 7, 17, 22, 30, 31, 34, 48, 62–66]	13	8.7 ± 12.3%	5.6% (8.8%)
**GFR reduction < 90 ml/min/1.73 m**^**2**^	Pediatric cohorts [4, 7, 22, 30, 31, 34, 62, 63, 66]	9	11.5 ± 15.9%	10.7% (11.0%)
**GFR reduction < 60 ml/min/1.73 m**^**2**^	Pediatric cohorts [17, 48, 64, 65]	4	4.4 ± 2.3%	4.8% (2.9%)
**GFR reduction**	Adult cohorts [38, 39, 67–69]	5	26.9 ± 10.6%	25.4% (17.7%)
**Proteinuria/albuminuria**	Pediatric cohorts [4, 7, 17, 22, 31, 34, 48, 62–65]	11	7.6 ± 5.8%	6.2% (9.4%)
	Adult cohorts [38, 39, 67–69]	5	29.0 ± 21.0%	43.0% (24.0%)
**Hypertension**	Pediatric cohorts [4, 7, 17, 22, 30, 31, 34, 48, 62–66]	13	7.1 ± 8.1%	5.0% (6.3%)
	Adult cohorts [38, 39, 67–69]	5	29.0 ± 22.6%	32.2% (14.6%)
**ABPM hypertension**	Pediatric cohorts [28, 37, 70–75]	8	28.0 ± 9.7%	31.1% (13.0%)
**ABPM masked hypertension**	Pediatric cohorts [28, 37, 70, 72, 74, 75]	6	14.3 ± 11.9%	19.9% (19.4%)

In square brackets: references

*Legend*: *GFR* glomerular filtration rate, *ABPM* ambulatory blood pressure monitoring

Risk factors which could affect outcome were analyzed in twelve of these studies, nine involving children [7, 17, 23, 31, 33, 34, 48, 64, 65] and three involving adults [38, 39, 69] (Table 6). The two most frequently analyzed risk factors in children were kidney length and the presence of an associated ipsilateral CAKUT. Compensatory enlargement has been described as a key parameter for normal kidney function [7] and, in general, subjects with a compensatory enlargement of the CSK and without ipsilateral CAKUT have a favorable outcome [7, 23, 31]. The strongest predictor of unfavorable outcome is the absence of compensatory enlargement of the CSK [7, 23, 38, 48, 64]. The presence of an ipsilateral CAKUT has also been described as a risk factor; however, it lost its significance at multivariate analysis in some cohorts [7, 17, 48]. A risk analysis of different CAKUT on the outcome in children with CSK is not available. The role of prematurity and low birth weight, which may impact nephrogenesis in terms of reduced nephron number [76], has also been analyzed in several studies [7, 17, 31, 48, 64]. The impact on kidney function in CSK appears limited in childhood, no studies being available in adults. Urinary tract infections in association with CSK could represent a risk factor, particularly if the infections are recurrent [7, 48, 64]. At present, a role for other studied risk factors does not appear to be relevant in the decline of GFR in children with CSK (Table 6). In adults, proteinuria and hypertension have been described as significant risk factors for decreased GFR [38, 39].

**Table 6:** Reported risk factors for an adverse renal outcome in subjects with CSK

			**Risk factors**																						
**Author, year**	**Age**	**Outcome**	**Absence of compensatory enlargement**	**CAKUT (ipsilateral)**	**UTI**	**Baseline creatinine**	**Baseline eGFR**	**Proteinuria**	**Hypertension**	**Hematuria**	**Age**	**Family History of CAKUT**	**Low Birth weight**	**Birth length**	**Prenatal diagnosis**	**Body mass index**	**Diabetes Mellitus**	**Uric Acid**	**RAS blockade**	**Duration of follow-up**	**Extrarenal anomalies**	**Gender**	**Phenotype**	**Prematurity**	**Side**
**Mansoor (2011) [**34**]**	**C**	**eGFR < 90 ml/min/1.73 m**^**2**^** 8/82 (9.7%)**		** + **																					
**Westland (2013)** **[**64]*	**C**	**KI 68/223 (31%)**	** + + **	** + + **	** + **						** + + **		** + **		** + **	ns						ns			ns
**Kolvek (2014)** [65**]**	**C**	**KI 9/27 (33.3%)**		** +**																					
		**eGFR < 60 ml/min/1.73 m**^**2**^ **3/27 (11.1%)**		+																					
**La Scola (2016) **[7]	**C**	**eGFR < 90 ml/min/1.73 m**^**2**^** 18/146 (12%)**	** + + **	** + **	** + **								ns			ns						ns		ns	ns
**Marzuillo (2017) **[31]**	**C**	**KI 12/306 (3.9%)**	ns	** + + **												ns						ns		ns	ns
**Urisarri (2018) **[17]	**C**	**eGFR < 60 ml/min/1.73 m**^**2**^** 3/128 (2.3%)**	ns	** + **							ns					ns						ns		ns	
**Poggiali (2019) **[48]	**C**	**KI 18/162 (11.1%)**	** + + **	** + **	** + + **	** + + **	** + **						** + **	ns							ns	ns	ns	ns	
		**eGFR < 60 ml/min/1.73 m**^**2**^** 9/162 (5.6%)**	ns	** + **	** + **	** + **	** + **						ns	** + **							ns	ns	ns	ns	
**Wang (2010) **[38]	**A**	**mGFR < 60 ml/min/1.73 m**^**2**^** 25/65 (38%)**	** + + **					** + + **	ns							ns				ns		ns			ns
**Basturk (2015) **[39]	**A**	**eGFR < 60 ml/min/1.73 m**^**2**^** 14/31 (45%)**	ns			ns	ns	** + **	** + **		ns					ns	** + **	** + **	ns	ns		ns			
**Xu (2019) **[69]	**A**	**eGFR < 60 ml/min/1.73 m**^**2**^** 30/118 (25.4%)**	** + **					** + **	** + **	** + **	ns	ns				ns		** + **			ns	ns			ns
		**eGFR < 30 ml/min/1.73 m**^**2**^** Prevalence NR**									ns	** + + **				ns					ns	ns			ns

White boxes correspond to not analyzed risk factors, + : significant at univariate analysis, +  + : significant at multivariate analysis when performed; *includes also cohorts described in reference 33; **includes also patients described in reference 23

*Legend*: *CSK* congenital solitary kidney, *C* children, *A* adults, *eGFR* estimated glomerular filtration rate, *KI* kidney injury, *mGFR* measured glomerular filtration rate, *NR* not reported, *ns* not significant, *CAKUT* congenital anomalies of kidney and urinary tract, *UTI* urinary tract infection, *RAS* renin angiotensin system

Based on the analyzed cohort studies (Tables 4 and 5) and on the reported risk factor analysis for GFR reduction (Table 6), we observe that two CSK populations exist. The first, in which there is no compensatory enlargement of the CSK and/or an additional ipsilateral CAKUT is present, is at increased risk for GFR reduction and chronic kidney disease (CKD) progression; the second, showing compensatory enlargement of the CSK and the absence of ipsilateral CAKUT, has a lesser risk for GFR reduction and CKD progression.

#### Statements/recommendations:

1. Children without compensatory enlargement of the CSK and/or additional ipsilateral CAKUT are at risk of GFR reduction and CKD progression (grade B).
2. Children showing a CSK with compensatory enlargement and the absence of ipsilateral CAKUT are at a lesser risk of GFR reduction and CKD progression (grade B).

### b) Is a child with CSK at risk of proteinuria?

Various studies evaluating kidney damage in patients with CSK have assessed the presence of proteinuria [7, 22, 23, 31, 34, 39, 48, 64, 67–69] and/or albuminuria [4, 17, 29, 33, 38, 62, 63, 65]. These studies, which had different inclusion criteria and follow-up periods, showed a variable prevalence of proteinuria/albuminuria (Table 4), both in children [4, 7, 17, 22, 23, 29, 31, 33, 34, 48, 62–65] and in adults [38, 39, 67–69]. In Table 5, we report the calculated weighted means, standard deviations, weighted medians, and IQRs for the prevalence of proteinuria/albuminuria, separately for children and adults. On the whole, the prevalence is higher than in the general pediatric population, in which it ranges between 0.0012 and 0.22% [77, 78]. The prevalence of albuminuria ranged between 3 and 24% in children [4, 17, 29, 33, 62, 63, 65] and was found in 48% of adults in one series [38]. In these series, the presence of proteinuria/albuminuria is reported as a categorical variable, so that their exact amount cannot be inferred. Proteinuria has been found to be higher in patients with ipsilateral CAKUT associated with CSK [17, 33, 48, 65]. Only one study evaluated the absence of kidney enlargement, baseline creatinine, and recurrent UTIs as risk factors for proteinuria and a positive correlation was found, while no correlation was found with low birth weight [48]. No studies have evaluated the role of proteinuria as a risk factor for GFR reduction in children with CSK. The use of antiproteinuric medications has only been reported in a few studies [33, 38, 64, 65]; however, no data on efficacy and safety are available.

#### Statements/recommendations:

1. The prevalence of proteinuria in children with a CSK is higher than in the normal pediatric population (Grade B).
2. Evaluation of proteinuria is warranted in every child with a CSK (grade B).

### c) Is a child with CSK at risk of developing hypertension?

A variable prevalence of hypertension has been documented by office BP recordings in children with CSK (Table 4) [4, 7, 17, 22, 23, 29–31, 33, 34, 48, 62–66]. In Table 5, we report the calculated weighted means, standard deviations, weighted medians, and IQRs for the prevalence of hypertension. The prevalence of hypertension in many cohorts was similar [4, 7, 17, 34, 48, 63] or even lower [22, 23, 30, 31, 62] than that expected in the general pediatric population [79], and was found to be higher in five out of 16 series [29, 33, 64–66]. In a meta-analysis performed on data from 1115 children with MCDK, six cases (0.5%) of hypertension were retrieved [80]. Few data are available on the age of onset of hypertension: children who were hypertensive or were taking kidney protective medications had a mean age of approximately 8 years at last follow-up in three series [29, 33, 64] and of 12 years at onset in one series [7]. In adults, a high prevalence of hypertension has been documented (Tables 4 and 5) [38, 39, 67–69]. This high prevalence may be biased by the fact that the series were hospital-based, selecting for the most severely affected patients with already established kidney disease [67]. A risk factor analysis of hypertension was performed in four out of 21 series [17, 33, 48, 65]. The most analyzed risk factor was the presence of an associated CAKUT, the results being conflicting.

#### Statements/Recommendations:

1. We recommend that office BP be measured in every child with a CSK (grade B).
2. At present, no clear risk factors for hypertension in children with a CSK have been demonstrated (grade C).

## Should ambulatory blood pressure monitoring be performed in children with CSK?

Ambulatory blood pressure monitoring (ABPM) is increasingly recognized as a useful tool in the diagnosis of hypertension, particularly because ABPM alone is able to detect white coat hypertension and masked hypertension [81, 82]; reference data for children are available from the age of 5 years [83, 84].

Ten studies evaluated BP by ABPM in a total number of 379 children with CSK [28, 32, 37, 70–75, 85] (Table 7). Five studies reported data on kidney length for a total of 136 patients and about 86% presented compensatory enlargement [28, 32, 70, 72, 85]. Six studies reported data on ipsilateral CAKUT, which were present in a minority of patients [28, 37, 70, 71, 75, 85], while in the study by Lubrano et al., 24 out of 38 had kidney scars [74]. Most of the children in these 10 studies had a GFR > 60 ml/min/1.73 m^2^, while approximately 22% of them had elevated office BP readings before ABPM. At ABPM, mean BP values were generally within the normal range, but were higher in children with CSK versus healthy controls [28, 71, 73, 75, 85] or versus children with other CAKUT [37]. The prevalence of ABPM-hypertension was reported in 8 out of 10 studies [28, 37, 70–75] (Table 7), with weighted mean prevalence of 27.9 ± 9.7% (Table 5); in the cohort of Lubrano et al., 82% of hypertensive children had kidney scars [74]. White coat hypertension was reported in six studies and was detected in a variable percentage of children (0–26%) [28, 37, 70, 72, 74, 75]. The weighted mean prevalence of masked hypertension, available from six studies, was 14.3 ± 11.9% (Table 5) [28, 37, 70, 72, 74, 75]. It has to be underlined that the high proportion of masked hypertension cases could have been overestimated, as it has previously been shown that many patients with masked hypertension become normotensive at a second ABPM [86]. On the other hand, the identification of masked hypertension is considered important, as some studies have shown a similar left ventricular mass index in subjects with either masked or sustained hypertension [81]. However, no studies relating ABPM levels to outcomes such as myocardial infarction or stroke are currently available for the pediatric population [81].

**Table 7:** Prevalence of hypertension and abnormal dipping in children with congenital solitary kidney at ambulatory blood pressure monitoring

Author, year	N	Age at ABPM (yrs)	Compensatory enlargement*	Ipsilateral CAKUT	eGFR < 90 ml/min/1.73 m^2^	OBP hypertension	ABPM hypertension	Masked hypertension	WCH	Abnormal dipping
Seeman T. (2001) [70]	25	7.8 (3.8–17.7)	21/25 (84%)	4/25 (16%)	2/25 Mean eGFR 55, 23/25 Mean eGFR ﻿110, range 85–159	8/25 (33%)	5/25 (20%)	1/25 (4%)	4/25 (16%)	5/25 (20%)
Mei-Zahav M. (2001) [85]	18	9.6 ± 3.9	18/18 (100%)	0/18	0/18	NR	NR	NR	NR	0/18
Seeman T. (2006) [28]	15	10.0 (4–17)	13/15 (87%)	0/15	0/15	5/15 (33%)	1/15 (7%)	0/15	4/15 (26%)	2/15 (14%)
Dursun H. (2007) [71]	44	8.3 ± 4.2	NR	0/44	0/44	NR	10/44 (23%)	NR	NR	13/44 (29.5%)
Westland R. (2014) [72]	28	12.5 ± 3.6	24/28 (86%)	NR	0/28	2/28 (7%)	7/28 (25%)	5/28 (18%)	0/28	8/28 (29%)
Tabel Y. (2015) [73]	44	10.9 ± 3.3	NR	NR	0/44	NR	19/44 (43%)	NR	NR	NR
Lubrano R. (2017) [74]	38	About 14.5	NR	Scars 24/38 (63%)	Mean eGFR 103.76 ± 46.49	11/38 (29%)	11/38 (29%)	0/38	0/38	NR
Zambaiti E. (2018) [32]	50	9.5 ± 4.2	39/50 (78.5%)	NR	Mean eGFR 80.6 ± 12.2	10/50 (20%)**	23/50 (46%)***	NR	NR	41/50 (82%)
La Scola C. (2020) [37]	81	11.8 ± 4.7	NR	9/50 (18%)	0/81	13/81 (16%)	27/81 (33.3%)	21/81 (25.9%)	7/81 (8.6%)	51/81 (64%)
Kasap-Demir B. (2021)[75]	36	11 ± 4.75	NR	0/36	1/36 (3%) Mean eGFR 127.3 ± 19.85	10/36 (28%)	7/36 (19%)	5/36 (14%)	8/36 (22%)	NR

^*^Kidney length ≥ 2DS for age or ≥ 95th percentile for height;**patients with elevated blood pressure defined as mean systolic and/or diastolic office blood pressure ≥ 90th but < 95th percentile; ***patients with either 24-h hypertension (ABPM values ≥ 95th percentile) or elevated blood pressure (ABPM values ≥ 90th but < 95th percentile)

*Legend*: *ABPM* ambulatory blood pressure monitoring, *CAKUT* congenital anomalies of kidney and urinary tract, *eGFR* estimated glomerular filtration rate, *OBP* office blood pressure, *WCH* white coat hypertension, *NR* not reported

We recommend that ABPM be performed in children with CSK and office BP > 95th percentile to determine whether sustained hypertension or white coat hypertension exists. As for the detection of masked hypertension, we believe that it should not be part of a routine screening in all children with a CSK and normal office BP, until more data become available on the clinical benefits of screening. On the other hand, ABPM should be considered in children in whom the CSK is associated with other high-risk conditions for hypertension, known for the general pediatric population, i.e., CKD grade ≥ II, a history of prematurity or obesity [87].

### Statements/recommendations:

1. We do not recommend routine ABPM in children with a CSK and normal office BP (grade C).
2. We recommend ABPM in children with a CSK, who are older than 5 years, with office BP > 95th percentile (grade C).
3. We recommend that ABPM be strongly considered in children in whom the CSK is associated with other high-risk conditions for hypertension (i.e., CKD grade ≥ II, a history of prematurity or obesity) (grade C).

## Can kidney protective medication be used safely in CSK?

Renin–angiotensin–aldosterone system (RAAS) inhibitors provide a clear kidney protective advantage in the progression of disease due to their ability to control hypertension and reduce proteinuria, whose proposed effects on the kidney include increased glomerulosclerosis, tubulointerstitial inflammation, and fibrosis, thereby contributing to progressive kidney function loss. Treatment with RAAS inhibitors, which decreases filtered proteins, also decreases the production of inflammatory cytokines and preserves kidney function [88]. Notwithstanding, in a retrospective study on children with bilateral hypodysplastic kidneys, treating or not with angiotensin converting enzyme inhibitors did not significantly modify the decline of kidney function [89]. Furthermore, the few studies that tested the impact of RAAS inhibitors on the function of solitary kidneys were mainly conducted in animals and only limited data have been reported for humans [90]. In a study involving 16 adults, no beneficial effects were found in terms of the progression of kidney failure [39]. Thus, this therapeutic approach in CSK remains controversial [90]. Notwithstanding, RAAS inhibitors in CSK can be indicated when signs of kidney damage progression are documented [3, 4, 21, 91] remembering that, in the infant, angiotensin inhibition can impair maturation of the kidney, exacerbate sodium wasting, and markedly reduce GFR [92]. Conversely, angiotensin inhibition is contraindicated in patients with arterial stenosis of the solitary kidney, due to the high risk of developing acute kidney injury [93]. An acute rise in serum creatinine, which should not exceed 25%, is expected after the start of RAAS inhibition [94]. Therefore, creatinine should be checked 3–4 weeks after starting treatment and the dose of RAAS inhibitors reduced or treatment withdrawn if creatinine increases > 25%. Moreover, the usual safety precautions for the use of these medications have to be considered.

### Statements/recommendations:

1. RAAS inhibitors should be used with caution in infants with a CSK (grade C).
2. RAAS inhibitors can be used beyond infancy to control hypertension and/or reduce proteinuria in children with CSK (grade C).
3. RAAS inhibitors must be avoided in patients with arterial stenosis of the solitary kidney (grade C).

## Which nutritional and lifestyle habits should be adopted for children with a CSK?

Children with a CSK showing compensatory enlargement and with normal BP should follow the same nutritional principles as the general pediatric population, and dietary recommendations issued for healthy children and adolescents [95] can serve as a guide; moreover, the importance of avoiding obesity and its long term consequences should be kept in mind [96]. The benefits of a low protein diet, which has often been advocated as useful in preserving kidney function in subjects with a reduced nephron number, have never been demonstrated in subjects with a solitary kidney [97]. On the other hand, it has to be remembered that in the Western world, protein intake is usually higher than recommended [98]. Therefore, avoiding the excessive protein intake so common in Western society is recommended in children with a CSK. As regards salt intake, various surveys and trials have demonstrated a very high intake by children in the Western world, and there is strong evidence to suggest that a high intake plays an important role in the genesis of hypertension and target organ damage [99]. For these reasons, excessive salt intake must be avoided in children with a CSK, remembering that table salt only represents approximately 10% of the daily intake, the greatest amount being contained in processed foods [99].

As in children with two kidneys, normal hydration should be guaranteed at all times, and in particular during sport activities, as a small-volume fluid intake, although not altering kidney function, is associated with an increased risk of urolithiasis and UTI [100].

Additional risk factors for kidney damage, including nephrotoxic drugs, should be avoided or minimized [21, 92]. In this respect, we suggest that acetaminophen be used to reduce fever and to relieve pain, thus avoiding the use of non-steroidal anti-inflammatory drugs.

### Statements/recommendations:

1. We recommend that in children with a CSK, protein intake should follow the dietary recommendations for children of the same age and sex, avoiding the excessive protein intake which is common in Western society (grade B).
2. We recommend that in children with a CSK, salt intake should follow the dietary recommendations for children of the same age and sex, avoiding the excessive salt intake which is common in Western society (grade B).
3. We recommend that dehydration be avoided, and regular fluid intake be encouraged, in particular during sport activities (grade C).
4. We recommend that the use of nephrotoxic drugs be avoided or minimized (grade C).

## Can sports be played without restraint by subjects with a CSK?

Whether or not children with a solitary kidney can play the same sports as their peers has been a matter of extensive debate. In that debate, it emerged that the majority of severe kidney injuries are not sport related, but due to road accidents or falls [101, 102]. Furthermore, various reports have highlighted that sport-related kidney injuries are very rare [101–105]. A literature search of papers published between 1966 and 2005 found an incidence of catastrophic sports-related kidney injury of 0.4 per 1 million children per year for all sports, the kidney being much less involved than other vital organs, such as the brain and spinal cord, which are also single organs [103]. In school athletes, kidney trauma was found to occur significantly less often than other organ-specific injuries: in the National Athletic Trainers’ Association registries 1995–1997, out of 4.4 million athlete exposures, 18 (0.07%) kidney injuries were identified, none of which led to genitourinary surgery or kidney loss [104]. A recent review of trauma data observed that “limited-contact” sports like skiing, snowboarding, sledding, biking, and horseback riding are more often associated with high-grade injury and kidney loss than contact sports like football [106]. The most common sports that can cause severe kidney injury are related to collision: sledding, skiing, snowboarding, cycling, rollerblading, and contact sports; equestrian activities can also be associated with kidney trauma [101–103].

As regards the use of protective equipment during sports, there is no evidence to suggest its efficacy in the prevention of kidney injury [107].

Policy statements have been issued on sports participation for children with solitary kidneys. In the latest American Academy of Pediatrics statement on sports in different medical conditions, it was observed that, for the majority of chronic health conditions, available evidence supports the participation of children and adolescents in most athletic activities [108]. For children with a solitary kidney, no restrictions on noncontact sports, and individual assessment for limited-contact, contact/collision sports was suggested. The use of protective equipment was encouraged. The Canadian Urological Association Best Practice Report on sports and the solitary kidney [107] supported the 2008 American Academy of Pediatrics policy statement. It also stated that caregivers should be informed about the sports that carry a higher risk of kidney injury, but also that they should be encouraged to remember that the activities most associated with high-grade kidney trauma have a much higher risk of head injury.

### Statements/recommendations:

1. We recommend that sport participation should not be restricted in children with CSK (grade B).
2. Caregivers and children should be informed that some sports, particularly if at risk of collision, (like cycling, sledding, downhill skiing/snowboarding, rollerblading, equestrian activities, and some contact sports) may carry a higher risk of kidney trauma than other activities (grade B) and that the use of flank protectors remains debated (grade C).

## What follow-up for children with a CSK?

Congenital solitary kidney has an impact on the entire life cycle. However, the understanding of its natural history is still incomplete: therefore, we provide our recommendations with the caveat that strong evidence on the risks of long-term complications is lacking.

As the absence of compensatory enlargement of the CSK and the presence of associated CAKUT appear important risk factors for progressive kidney damage, we propose that, following the assessment previously described, the follow-up schedule be based on risk stratification, as follows:

low risk: kidney length > 50th percentile in the first 2 years of life and ≥ 95th percentile thereafter, and absence of ipsilateral CAKUT

medium risk: CSK without compensatory enlargement, and/or with an ipsilateral CAKUT

high risk: decreased eGFR (i.e., mean eGFR for age − 1 SD in children younger than 2 years, < 90 ml/min/1.73 m^2^ in children older than 2 years) and/or proteinuria, and/or hypertension.

In our opinion, children at low risk can be followed by general pediatricians (provided it is feasible on the basis of the local health care system), while children at medium risk, who need specialistic surveillance, should be under the care of a pediatric nephrologist, and those at high risk should be followed in pediatric nephrology units. The type and timing of the recommended check-ups are reported in Table 8. In all the risk classes, females must undergo an abdominopelvic US after thelarche and before menarche, for the evaluation of the genital apparatus. Moreover, in all the risk classes, follow-up must be continued during adolescence. Finally, the transition of patients from pediatric to adult healthcare providers has to be accurately planned, as it represents a critical period in terms of maintaining kidney function [92].

**Table 8:** Opinion-based recommendations for follow-up in children and adolescents with congenital solitary kidney

	Low risk*	Medium risk*		High risk*
		**Without CAKUT**	**With ipsilateral CAKUT**	
**Setting**	Primary pediatric care^1^	Pediatric nephrologist^1^/pediatric nephrology unit		Pediatric nephrology unit
**Ultrasound**^**2**^	Yearly until 3 years of age, then every 5 years	Yearly until 3 years of age, then every 3 to 5 years	Further work-up depending on additional ipsilateral CAKUT findings	According to kidney function and clinical data
**Proteinuria by urinalysis**^**3**^	Yearly until 3 years of age, then every 5 years	Yearly		
**Office Blood pressure**	Yearly ≥ 3 years	Yearly		
**Serum creatinine/eGFR**	Not necessary	Yearly		
**Abdominopelvic ultrasound in girls**	Between thelarche and menarche	Between thelarche and menarche		Between thelarche and menarche

^*^Risk stratification:

- low risk: kidney length > 50th pct in the first 2 years of life and ≥ 95th pct thereafter, and absence of ipsilateral CAKUT

- medium risk: CSK without compensatory enlargement, and/or with an ipsilateral CAKUT

- high risk: decreased eGFR (i.e., mean eGFR for age -1 SD in children younger than 2 years, < 90 ml/min per 1.73 m^2^ in children older than 2 years) and/or proteinuria, and/or hypertension

1. As feasible according to the organization of the local health care system; 2. With measurement of kidney length/size; 3. If proteinuria is detected, quantification by urine protein/urine creatinine should be performed in the second morning urine sample, remembering that normal values are < 0.5 mg/mg until two years of age and < 0.2 thereafter

*Legend*: *CAKUT* congenital anomalies of kidney and urinary tract, *eGFR* estimated glomerular filtration rate

### Statements/recommendations:

1. We recommend that all children with a CSK be followed until adulthood as outlined in Table 8, according to risk stratification (grade B).
2. We suggest that children at low risk should be followed by general pediatricians, provided it is feasible on the basis of the local health care system, while children at medium risk should be under the care of a pediatric nephrologist and children at high risk be followed in pediatric nephrology units (grade C).

## Health benefits, limitations of our recommendations and future perspectives

This consensus statement provides guidance on the initial diagnostic work-up, the nutritional and lifestyle habits, and the follow-up of children with CSK.

We believe that health benefits are obtained by a reduction of the number of VCUG and nuclear scans performed, therefore decreasing radiation exposure and financial costs. Another benefit may be gained by restricting the prescription of laboratory blood testing.

Our recommendations are not without limitations. First, available evidence to support most of these recommendations comes from retrospective cohort studies, because randomized clinical trials or prospective studies with consistent and good-quality patient-oriented evidence are lacking. Second, this is a consensus statement developed by pediatric nephrologists; other specialists may have different opinions. However, these recommendations can be used for comparison with other available indications and protocols, as a worldwide consensus in this area is lacking.

The authors of these recommendations have found some gaps in our knowledge about CSK:

- first, US growth nomograms specific for CSK on large cohorts are lacking and should be elaborated;
- second, further studies are needed to validate a risk stratification model applicable early in life, to tailor follow-up accordingly;
- third, the presence, degree, and time course of hyperfiltration in human CSK and its influence on subsequent kidney injury should be studied.

## Supplementary Information

Below is the link to the electronic supplementary material.

Supplementary file1 (DOCX 36 KB)

## Data Availability

Data obtained from the literature.

## Code availability

NA.
