# Supplementary material for: Management of the congenital solitary kidney: consensus recommendations of the Italian Society of Pediatric Nephrology
Source: Pediatr Nephrol. 2022 Jun 17;37(9):2185–207. doi: 10.1007/s00467-022-05528-y (PMC9307550; doi:10.1007/s00467-022-05528-y)
Supplement: Supplementary file 1 — Supplementary file1 (DOCX 36 KB) [file 467_2022_5528_MOESM1_ESM.docx]

**Supplementary list of the 72 articles included**

|  | Year | First Authors | Title | Study design |
| --- | --- | --- | --- | --- |
| 1 | 1992 | Argueso LR | Prognosis of patients with unilateral renal agenesis. Pediatr Nephrol 6:412–416. https://doi.org/10.1007/BF00873996 | Retrospective cohort study |
| 2 | 1997 | Maluf NSR | On the enlargement of the normal congenitally solitary kidney | Case report |
| 3 | 2001 | Mei-Zahav M | Ambulatory blood pressure monitoring in children with a solitary kidney - A comparison between unilateral renal agenesis and uninephrectomy. | Cross sectional study |
| 4 | 2001 | Seeman T | Ambulatory blood pressure monitoring in children with unilateral multicystic dysplastic kidney. | Cross sectional study |
| 5 | 2002 | Abidari JM | Serial followup of the contralateral renal size in children with multicystic dysplastic kidney | Retrospective cohort study |
| 6 | 2005 | Wiesel A | Prenatal detection of congenital renal malformations by fetal ultrasonographic examination: An analysis of 709,030 births in 12 European countries. | Registry based observational study |
| 7 | 2005 | Shaheen IS | Multicystic dysplastic kidney and pelviureteric junction obstruction. | Case report |
| 8 | 2005 | Dursun H | Associated anomalies in children with congenital solitary functioning kidney. | Retrospective cohort study |
| 9 | 2005 | Narchi H | Risk of hypertension with multicystic kidney disease: A systematic review. | Systematic review |
| 10 | 2005 | Johnson B | A need for reevaluation of sports participation recommendations for children with a solitary kidney. | Registry based observational study |
| 11 | 2006 | Seeman T | Blood pressure, renal function, and proteinuria in children with unilateral renal agenesis. | Cross sectional study |
| 12 | 2006 | Grinsell MM | Single kidney and sports participation: Perception versus reality. | Survey |
| 13 | 2007 | Woolf AS | Unilateral renal agenesis and the congenital solitary functioning kidney: Developmental, genetic and clinical perspectives. | Systematic Review |
| 14 | 2007 | Dursun H | Ambulatory blood pressure monitoring and renal functions in children with a solitary kidney. | Cross sectional study |
| 15 | 2008 | Vu KH | Renal outcome of children with one functioning kidney from birth. A study of 99 patients and a review of the literature. | Retrospective cohort study |
| 16 | 2008 | Weinstein A | Simple multicystic dysplastic kidney disease: End points for subspecialty follow-up | Retrospective cohort study |
| 17 | 2008 | Schreuder MF | Hypertension and microalbuminuria in children with congenital solitary kidneys. J | Retrospective cohort study |
| 18 | 2008 | Brophy RH | Kidney injuries in professional American football: Implications for management of an athlete with 1 functioning kidney. | Case series |
| 19 | 2008 | Rice SG | Medical conditions affecting sports participation | Clinical Report |
| 20 | 2009 | Schreuder MF | Unilateral multicystic dysplastic kidney: A meta-analysis of observational studies on the incidence, associated urinary tract malformations and the contralateral kidney. | Metanalysis |
| 21 | 2009 | Sanna-Cherchi S | Renal outcome in patients with congenital anomalies of the kidney and urinary tract. | Retrospective cohort study |
| 22 | 2009 | Hegde S | Renal agenesis and unilateral nephrectomy: What are the risks of living with a single kidney? | Review |
| 23 | 2009 | Chevalier R | When is one kidney not enough? | Editorial comment |
| 24 | 2010 | Oh KY | Prenatal diagnosis of renal developmental anomalies associated with an empty renal fossa | Review |
| 25 | 2010 | Wang Y | Analysis of factors associated with renal function in Chinese adults with congenital solitary kidney | Retrospective cohort study |
| 26 | 2011 | Corbani V | Congenital solitary functioning kidneys: Which ones warrant follow-up into adult life? | Editorial comment |
| 27 | 2011 | Westland R | Renal injury in children with a solitary functioning kidney-the KIMONO study. | Retrospective cohort study |
| 28 | 2011 | Mansoor | O Long-term risk of chronic kidney disease in unilateral multicystic dysplastic kidney. | Retrospective cohort study |
| 29 | 2011 | Abou Jaoudé P | Congenital versus acquired solitary kidney: Is the difference relevant? | Retrospective cohort study |
| 30 | 2012 | Van Vuuren SH | Compensatory enlargement of a solitary functioning kidney during fetal development. | Retrospective cohort study |
| 31 | 2012 | Krill A | Abdominopelvic ultrasound: A cost-effective way to diagnose solitary kidney | Retrospective cohort study |
| 32 | 2012 | Hayes WN | Unilateral multicystic dysplastic kidney: Does initial size matter? | Retrospective cohort study |
| 33 | 2012 | Stefanowicz | Renal function and solitary kidney disease: Wilms tumour survivors versus patients with unilateral renal agenesis. Kidney Blood Press | Retrospective cohort study |
| 34 | 2012 | Grinsell MM | Sport-related kidney injury among high school athletes | Retrospective cohort study |
| 35 | 2013 | Westland R | Unilateral renal agenesis: A systematic review on associated anomalies and renal injury | Systematic review |
| 36 | 2013 | Cachat F | Microalbuminuria and hyperfiltration in subjects with nephro-urological disorders. | Retrospective cohort study |
| 37 | 2013 | Westland R | Risk factors for renal injury in children with a solitary functioning kidney. | Retrospective cohort study |
| 38 | 2014 | Westland R | Clinical implications of the solitary functioning kidney. | Review |
| 39 | 2014 | Whittam BM | Ultrasound diagnosis of multicystic dysplastic kidney: Is a confirmatory nuclear medicine scan necessary? | Cross sectional study |
| 40 | 2014 | Shirzai A | Is microalbuminuria a risk factor for hypertension in children with solitary kidney? | Retrospective cohort study |
| 41 | 2014 | Kolvek G | Solitary functioning kidney in children - A follow-up study. | Prospective cohort study |
| 42 | 2014 | Siomou E | Growth and function in childhood of a normal solitary kidney from birth or from early infancy. | Retrospective cohort study |
| 43 | 2014 | Westland R | Ambulatory blood pressure monitoring is recommended in the clinical management of children with a solitary functioning kidney | Cross sectional study |
| 44 | 2015 | Basturk T | Renal damage frequency in patients with solitary kidney and factors that affect progression. | Retrospective cohort study |
| 45 | 2015 | Ross I | Sonographic assessment of the effect of vesicoureteral reflux and urinary tract infections on growth of the pediatric solitary kidney | Retrospective cohort study |
| 46 | 2015 | Tabel Y | Evaluation of hypertension by ambulatory blood pressure monitoring in children with solitary kidney. | Cross sectional study |
| 47 | 2016 | Groen in ’t Woud S | Maternal risk factors involved in specific congenital anomalies of the kidney and urinary tract: A case–control study. | Case control study |
| 48 | 2016 | La Scola C | Congenital Solitary Kidney in Children: Size Matters. | Retrospective cohort study |
| 49 | 2017 | Heidet L | Targeted exome sequencing identifies PBX1 as involved in monogenic congenital anomalies of the kidney and urinary tract | Translational Research, cross sectional |
| 50 | 2017 | Marzuillo P | Outcomes of a Cohort of Prenatally Diagnosed and Early Enrolled Patients with Congenital Solitary Functioning Kidney | Retrospective cohort study |
| 51 | 2017 | Wu H | Identification of 8 Novel Mutations in Nephrogenesis-Related Genes in Chinese Han Patients with Unilateral Renal Agenesis | Translational Research, cross-sectional |
| 52 | 2017 | Lubrano R | Evolution of blood pressure in children with congenital and acquired solitary functioning kidney | Retrospective cohort study |
| 53 | 2017 | Simeoni M | Current evidence on the use of anti-raas agents in congenital or acquired solitary kidney. | Review |
| 54 | 2017 | Papagiannopoulos D | Revisiting Sports Precautions in Children With Solitary Kidneys and Congenital Anomalies of the Kidney and Urinary Tract. | Systematic Review |
| 55 | 2018 | Acién P | The presentation and management of complex female genital malformations | Systematic Review |
| 56 | 2018 | Schreuder MF | Life with one kidney | Review |
| 57 | 2018 | Friedman MA | Screening for Mullerian anomalies in patients with unilateral renal agenesis: Leveraging early detection to prevent complications | Systematic Review |
| 58 | 2018 | Urisarri A | Retrospective study to identify risk factors for chronic kidney disease in children with congenital solitary functioning kidney detected by neonatal renal ultrasound screening. | Retrospective cohort study |
| 59 | 2019 | Cochat P | Towards adulthood with a solitary kidney. | Review |
| 60 | 2019 | Marzuillo P | Congenital solitary kidney size at birth could predict reduced eGFR levels later in life. | Retrospective cohort study |
| 61 | 2019 | Zambaiti E | Correlation between hypertrophy and risk of hypertension in congenital solitary functioning kidney | Retrospective cohort study |
| 62 | 2019 | Brown C | Knowledge of vesicoureteral reflux obtained by screening voiding cystourethrogram in children with multicystic dysplastic kidney does not change patient management or prevent febrile urinary tract infection. | Retrospective cohort study |
| 63 | 2019 | Poggiali IV | A clinical predictive model of renal injury in children with congenital solitary functioning kidney. | Retrospective cohort study |
| 64 | 2019 | Yamamoto K | Necessity of performing voiding cystourethrography for children with unilateral multicystic dysplastic kidney | Cross-sectional study |
| 65 | 2019 | Ishiwa S | Association between the clinical presentation of congenital anomalies of the kidney and urinary tract (CAKUT) and gene mutations: an analysis of 66 patients at a single institution | Retrospective cohort study |
| 66 | 2019 | Xu Q | The clinical characteristics of Chinese patients with unilateral renal agenesis. | Prospective cohort study |
| 67 | 2019 | Psooy K | Sports and the solitary kidney - What primary caregivers of a young child with a single kidney should know (2019 update). | Guideline |
| 68 | 2020 | La Scola C | Born with a solitary kidney: at risk of hypertension. | Cross-Sectional multicenter study. |
| 69 | 2020 | Blachman-Braun R | Voiding Cystourethrogram in Children With Unilateral Multicystic Dysplastic Kidney: Is It Still necessary? | Retrospective cohort study |
| 70 | 2020 | La Scola C | Effect of Body Mass Index on Estimated Glomerular Filtration Rate Levels in Children With Congenital Solitary Kidney: A Cross-Sectional Multicenter Study. | Cross-Sectional multicenter study. |
| 71 | 2021 | Groen in ’t Woud S | Clinical Management of Children with a Congenital Solitary Functioning Kidney: Overview and Recommendations. | Review |
| 72 | 2021 | Kasap-Demir J | Cardiovascular risk assessment in children and adolescents with congenital solitary kidneys. | Cross sectional study |

**References**

1. Argueso LR, Ritchey ML, Boyle ET Jr, Milliner DS, Bergstralh EJ, Kramer SA. (1992) Prognosis of patients with unilateral renal agenesis. Pediatr Nephrol 6:412–416. https://doi.org/10.1007/BF00873996
2. Maluf NSR (1997) On the enlargement of the normal congenitally solitary kidney. Br J Urol 79:836–841. https://doi.org/10.1046/j.1464-410x.1997.00215.x
3. Mei-Zahav M, Korzets Z, Cohen I, Kessler O, et al (2001) Ambulatory blood pressure monitoring in children with a solitary kidney - A comparison between unilateral renal agenesis and uninephrectomy. Blood Press Monit 6:263–267. https://doi.org/10.1097/00126097-200110000-00007
4. Seeman T, John U, Bláhová K, Vondrichová H, et al. (2001) Ambulatory blood pressure monitoring in children with unilateral multicystic dysplastic kidney. Eur J Pediatr. 160(2):78-83. https://doi.org/10.1007/s004310000579
5. Abidari JM, Park KH, Kennedy WA, Shortliffe LD (2002) Serial followup of the contralateral renal size in children with multicystic dysplastic kidney. J Urol 168:1821–1825. https://doi.org/10.1016/s0022-5347(05)64422-9
6. Wiesel A, Queisser-Luft A, Clementi M, Bianca S, et al (2005) Prenatal detection of congenital renal malformations by fetal ultrasonographic examination: An analysis of 709,030 births in 12 European countries. Eur J Med Genet 48:131–144. https://doi.org/10.1016/j.ejmg.2005.02.003
7. Shaheen IS, Watson AR, Broderick N, Rance C (2005) Multicystic dysplastic kidney and pelviureteric junction obstruction. Pediatr Surg Int 21:282–284. https://doi.org/10.1007/S00383-004-1303-7
8. Dursun H, Bayazit AK, Büyükçelik M, Soran M, et al (2005) Associated anomalies in children with congenital solitary functioning kidney. Pediatr Surg Int 21:456–459. https://doi.org/10.1007/s00383-005-1408-7
9. Narchi H (2005) Risk of hypertension with multicystic kidney disease: A systematic review. Arch Dis Child 90:921–924. https://doi.org/10.1136/adc.2005.075333
10. Johnson B, Christensen C, Dirusso S, Choudhury M, et al (2005) A need for reevaluation of sports participation recommendations for children with a solitary kidney. J Urol 174:686–689. https://doi.org/10.1097/01.ju.0000164719.91332.42
11. Seeman T, Patzer L, John U, Dušek J, et al (2006) Blood pressure, renal function, and proteinuria in children with unilateral renal agenesis. Kidney Blood Press Res 29:210–215. https://doi.org/10.1159/000095735
12. Grinsell MM, Showalter S, Gordon KA, Norwood VF (2006) Single kidney and sports participation: Perception versus reality. Pediatrics 118:1019–1027. https://doi.org/10.1542/peds.2006-0663
13. Woolf AS, Hillman KA (2007) Unilateral renal agenesis and the congenital solitary functioning kidney: Developmental, genetic and clinical perspectives. BJU Int 99:17–21. https://doi.org/10.1111/j.1464-410X.2006.06504.x
14. Dursun H, Bayazit AK, Cengiz N, Seydaoglu G, et al (2007) Ambulatory blood pressure monitoring and renal functions in children with a solitary kidney. Pediatr Nephrol 22:559–564. https://doi.org/10.1007/s00467-006-0389-7
15. Vu KH, Van Dyck M, Daniels H, Proesmans W (2008) Renal outcome of children with one functioning kidney from birth. A study of 99 patients and a review of the literature. Eur J Pediatr 167:885–890. https://doi.org/10.1007/s00431-007-0612-y
16. Weinstein A, Goodman TR, Iragorri S (2008) Simple multicystic dysplastic kidney disease: End points for subspecialty follow-up. Pediatr Nephrol 23:111–116. https://doi.org/10.1007/s00467-007-0635-7
17. Schreuder MF, Langemeijer ME, Bökenkamp A, Delemarre-Van de Waal HA and Van Wijk JAE (2008) Hypertension and microalbuminuria in children with congenital solitary kidneys. J Paediatr Child Health 44:363–368. https://doi.org/10.1111/j.1440-1754.2008.01315.x
18. Brophy RH, Gamradt SC, Barnes RP, Powell JW, et al (2008) Kidney injuries in professional American football: Implications for management of an athlete with 1 functioning kidney. Am J Sports Med 36:85–90. https://doi.org/10.1177/0363546507308940
19. Rice SG, American Academy of Pediatrics Council on Sports Medicine and Fitness (2008) Medical conditions affecting sports participation. Pediatrics 121:841–848. https://doi.org/10.1542/peds.2008-0080
20. Schreuder MF, Westland R, Van Wijk JAE (2009) Unilateral multicystic dysplastic kidney: A meta-analysis of observational studies on the incidence, associated urinary tract malformations and the contralateral kidney. Nephrol Dial Transplant 24:1810–1818. https://doi.org/10.1093/ndt/gfn777
21. Sanna-Cherchi S, Ravani P, Corbani V, Parodi S, Haupt R, et al (2009) Renal outcome in patients with congenital anomalies of the kidney and urinary tract. Kidney Int 76:528–533. https://doi.org/10.1038/ki.2009.220
22. Hegde S, Coulthard MG (2009) Renal agenesis and unilateral nephrectomy: What are the risks of living with a single kidney? Pediatr Nephrol 24:439–446. https://doi.org/10.1007/s00467-008-0924-9
23. Chevalier RL (2009) When is one kidney not enough? Kidney Int 76:475–477. https://doi.org/10.1038/ki.2009.244
24. Oh KY, Holznagel DE, Ameli JR, Sohaey R (2010) Prenatal diagnosis of renal developmental anomalies associated with an empty renal fossa. Ultrasound Q 26:233–240. https://doi.org/10.1097/RUQ.0b013e3181f573fd
25. Wang Y, Wang Z, Wang W, Ren H, et al (2010) Analysis of factors associated with renal function in Chinese adults with congenital solitary kidney. Intern Med 49:2203–2209. https://doi.org/10.2169/internalmedicine.49.3742
26. Corbani V, Ghiggeri GM, Sanna-Cherchi S (2011) Congenital solitary functioning kidneys: Which ones warrant follow-up into adult life? Nephrol Dial Transplant 26:1458–1460. https://doi.org/10.1093/ndt/gfr145
27. Westland R, Schreuder MF, Bökenkamp A, Spreeuwenberg MD, and van Wijk JAE (2011) Renal injury in children with a solitary functioning kidney-the KIMONO study. Nephrol Dial Transplant 26:1533–1541. https://doi.org/10.1093/ndt/gfq844
28. Mansoor O, Chandar J, Rodriguez MM, Abitbol CL, et al (2011) Long-term risk of chronic kidney disease in unilateral multicystic dysplastic kidney. Pediatr Nephrol 26:597–603. https://doi.org/10.1007/s00467-010-1746-0
29. Abou Jaoudé P, Dubourg L, Bacchetta J, Berthiller J, et al (2011) Congenital versus acquired solitary kidney: Is the difference relevant? Nephrol Dial Transplant 26:2188–2194. https://doi.org/10.1093/ndt/gfq659
30. Van Vuuren SH, Van Der Doef R, Cohen-Overbeek TE, Goldschmeding R, et al (2012) Compensatory enlargement of a solitary functioning kidney during fetal development. Ultrasound Obstet Gynecol 40:665–668. https://doi.org/10.1002/uog.11168
31. Krill A, Cubillos J, Gitlin J, Palmer LS (2012) Abdominopelvic ultrasound: A cost-effective way to diagnose solitary kidney. J Urol 187:2201–2204. https://doi.org/10.1016/j.juro.2012.01.129
32. Hayes WN, Watson AR (2012) Unilateral multicystic dysplastic kidney: Does initial size matter? Pediatr Nephrol 27:1335–1340. https://doi.org/10.1007/s00467-012-2141-9
33. Stefanowicz J, Owczuk R, Kałużyńska B, Aleksandrowicz E, et al (2012) Renal function and solitary kidney disease: Wilms tumour survivors versus patients with unilateral renal agenesis. Kidney Blood Press Res 35:174–181. https://doi.org/10.1159/000332083
34. Grinsell MM, Butz K, Gurka MJ, Gurka KK, Norwood V (2012) Sport-related kidney injury among high school athletes. Pediatrics 130:e40-45. https://doi.org/10.1542/peds.2011-2082
35. Westland R, Schreuder MF, Ket JCF, Van Wijk JAE (2013) Unilateral renal agenesis: A systematic review on associated anomalies and renal injury. Nephrol Dial Transplant 28:1844–1855. https://doi.org/10.1093/ndt/gft012
36. Cachat F, Combescure C, Chehade H, Zeier G, et al (2013) Microalbuminuria and hyperfiltration in subjects with nephro-urological disorders. Nephrol Dial Transplant 28:386–391. https://doi.org/10.1093/ndt/gfs494
37. Westland R, Kurvers RAJ, Van Wijk JAE, Schreuder MF (2013) Risk factors for renal injury in children with a solitary functioning kidney. Pediatrics 131:e478-485. https://doi.org/10.1542/peds.2012-2088
38. Westland R, Schreuder MF, van Goudoever JB, Sanna-Cherchi S, van Wijk JAE (2014) Clinical implications of the solitary functioning kidney. Clin J Am Soc Nephrol 9:978–986. https://doi.org/10.2215/CJN.08900813
39. Whittam BM, Calaway A, Szymanski KM, Carroll AE , et al (2014) Ultrasound diagnosis of multicystic dysplastic kidney: Is a confirmatory nuclear medicine scan necessary? J Pediatr Urol 10:1059–1062. https://doi.org/10.1016/j.jpurol.2014.03.011
40. Shirzai A, Yildiz N, Biyikli N, Ustunsoy S, et al (2014) Is microalbuminuria a risk factor for hypertension in children with solitary kidney? Pediatr Nephrol 29:283–288. https://doi.org/10.1007/s00467-013-2641-2
41. Kolvek G, Podracka L, Rosenberger J, Stewart RE, et al (2014) Solitary functioning kidney in children - A follow-up study. Kidney Blood Press Res 39:272–278. https://doi.org/10.1159/000355804
42. Siomou E, Giapros V, Papadopoulou F, Pavlou M, et al (2014) Growth and function in childhood of a normal solitary kidney from birth or from early infancy. Pediatr Nephrol 29:249–256. https://doi.org/10.1007/s00467-013-2623-4
43. Westland R, Schreuder MF, van der Lof DF, Vermeulen A, et al (2014) Ambulatory blood pressure monitoring is recommended in the clinical management of children with a solitary functioning kidney. Pediatr Nephrol 29:2205–2211. https://doi.org/10.1007/s00467-014-2853-0
44. Basturk T, Koc Y, Ucar Z, Sakaci E, et al (2015) Renal damage frequency in patients with solitary kidney and factors that affect progression. Int J Nephrol. https://doi.org/10.1155/2015/876907
45. Ross I, Ahn HJ, Roelof B, Barber T, et al (2015) Sonographic assessment of the effect of vesicoureteral reflux and urinary tract infections on growth of the pediatric solitary kidney. J Pediatr Urol 11:145.e1-145.e6. https://doi.org/10.1016/j.jpurol.2015.02.012
46. Tabel Y, Aksoy Ö, Elmas AT, Çelik SF (2015) Evaluation of hypertension by ambulatory blood pressure monitoring in children with solitary kidney. Blood Press 24:119–123. https://doi.org/10.3109/08037051.2014.992194
47. Groen in ’t Woud S, Renkema KY, Schreuder MF, Wijers CHW, et al (2016) Maternal risk factors involved in specific congenital anomalies of the kidney and urinary tract: A case–control study. Birth Defects Res Part A - Clin Mol Teratol 106:596–603. https://doi.org/10.1002/bdra.23500
48. La Scola C, Ammenti A, Puccio G, Lega ML, et al (2016) Congenital Solitary Kidney in Children: Size Matters. J Urol 196:1250–1256. https://doi.org/10.1016/j.juro.2016.03.173
49. Heidet L, Morinière V, Henry C, De Tomasi L, et al (2017) Targeted exome sequencing identifies PBX1 as involved in monogenic congenital anomalies of the kidney and urinary tract. J Am Soc Nephrol 28:2901–2914. https://doi.org/10.1681/ASN.2017010043
50. Marzuillo P, Guarino S, Grandone A, Di Somma A, et al (2017) Outcomes of a Cohort of Prenatally Diagnosed and Early Enrolled Patients with Congenital Solitary Functioning Kidney. J Urol 198:1153–1158. https://doi.org/10.1016/j.juro.2017.05.076
51. Wu H, Xu Q, Xie J, Ma J, et al (2017) Identification of 8 Novel Mutations in Nephrogenesis-Related Genes in Chinese Han Patients with Unilateral Renal Agenesis. Am J Nephrol 46:55–63. https://doi.org/10.1159/000477590
52. Lubrano R, Gentile I, Falsaperla R, Vitaliti G, et al (2017) Evolution of blood pressure in children with congenital and acquired solitary functioning kidney. Ital J Pediatr 43:1–6. https://doi.org/10.1186/s13052-017-0359-7
53. Simeoni M, Armeni A, Summaria C, Cerantonio A, Fuiano G. et al (2017) Current evidence on the use of anti-raas agents in congenital or acquired solitary kidney. Ren Fail 39:660–670. https://doi.org/10.1080/0886022X.2017.1361840
54. Papagiannopoulos D, Gong E (2017) Revisiting Sports Precautions in Children With Solitary Kidneys and Congenital Anomalies of the Kidney and Urinary Tract. Urology 101:9–14. https://doi.org/10.1016/j.urology.2016.11.025
55. Acién P, Acién M (2016) The presentation and management of complex female genital malformations. Hum Reprod Update 22:48–69. https://doi.org/10.1093/humupd/dmv048
56. Schreuder MF (2018) Life with one kidney. Pediatr Nephrol 33:595–604. https://doi.org/10.1007/s00467-017-3686-4
57. Friedman MA, Aguilar L, Heyward Q, Wheeler C, Caldamone A (2018) Screening for Mullerian anomalies in patients with unilateral renal agenesis: Leveraging early detection to prevent complications. J Pediatr Urol 14:144–149. https://doi.org/10.1016/j.jpurol.2018.01.011
58. Urisarri A, Gil M, Mandiá N, Aldamiz-Echevarría L, et al (2018) Retrospective study to identify risk factors for chronic kidney disease in children with congenital solitary functioning kidney detected by neonatal renal ultrasound screening. Med 97:e11819. https://doi.org/10.1097/MD.0000000000011819
59. Cochat P, Febvey O, Bacchetta J, Bérard E, et al (2019) Towards adulthood with a solitary kidney. Pediatr Nephrol 34:2311–2323. https://doi.org/10.1007/s00467-018-4085-1
60. Marzuillo P, Guarino S, Grandone A, Di Somma A, et al (2019) Congenital solitary kidney size at birth could predict reduced eGFR levels later in life. J Perinatol 39:129–134. https://doi.org/10.1038/s41372-018-0260-2
61. Zambaiti E, Sergio M, Baldanza F, Corrado C, et al (2019) Correlation between hypertrophy and risk of hypertension in congenital solitary functioning kidney. Pediatr Surg Int 35:167–174. https://doi.org/10.1007/s00383-018-4389-z
62. Brown C, McLeod D, Ching C (2019) Knowledge of vesicoureteral reflux obtained by screening voiding cystourethrogram in children with multicystic dysplastic kidney does not change patient management or prevent febrile urinary tract infection. J Pediatr Urol 15:267.e1-267.e5. https://doi.org/10.1016/j.jpurol.2019.03.013
63. Poggiali IV, Simões e Silva AC, Vasconcelos MA, Dias CS, et al (2019) A clinical predictive model of renal injury in children with congenital solitary functioning kidney. Pediatr Nephrol 34:465–474. https://doi.org/10.1007/s00467-018-4111-3
64. Yamamoto K, Kamei K, Sato M, Ogura M, et al. Necessity of performing voiding cystourethrography for children with unilateral multicystic dysplastic kidney. Pediatr Nephrol. 2019 Feb;34(2):295-299. doi: 10.1007/s00467-018-4079-z. Epub 2018 Sep 25. PMID: 30255447.
65. Ishiwa S, Sato M, Morisada N, Nishi K, Kanamori T, et al (2019) Association between the clinical presentation of congenital anomalies of the kidney and urinary tract (CAKUT) and gene mutations: an analysis of 66 patients at a single institution. Pediatr Nephrol 34:1457–1464. https://doi.org/10.1007/s00467-019-04230-w
66. Xu Q, Wu H, Zhou L, Xie J, et al (2019) The clinical characteristics of Chinese patients with unilateral renal agenesis. Clin Exp Nephrol 23:792–798. https://doi.org/10.1007/s10157-019-01704-x
67. Psooy K, Franc-Guimond J, Kiddoo D, Lorenzo A, MacLellan D (2019) Canadian Urological Association best practice report: Sports and the solitary kidney - What primary caregivers of a young child with a single kidney should know (2019 update). Can Urol Assoc J 13:315–317. https://doi.org/10.5489/cuaj.6118
68. La Scola C, Marra G, Ammenti A, Pasini A, et al (2020) Born with a solitary kidney: at risk of hypertension. Pediatr Nephrol 35:1483–1490. https://doi.org/10.1007/s00467-020-04535-1
69. Blachman-Braun R, Camp MM, Becerra MF, Christian G, et al (2020) Voiding Cystourethrogram in Children With Unilateral Multicystic Dysplastic Kidney: Is It Still necessary? Urology 139:156–160. https://doi.org/10.1016/j.urology.2020.02.005
70. La Scola C, Guarino S, Pasini A, Capalbo D, et al (2020) Effect of Body Mass Index on Estimated Glomerular Filtration Rate Levels in Children With Congenital Solitary Kidney: A Cross-Sectional Multicenter Study. J Ren Nutr 30:261–267. https://doi.org/10.1053/j.jrn.2019.07.003
71. Groen in ’t Woud S, Westland R, Feitz WFJ, Roeleveld N, et al (2021) Clinical Management of Children with a Congenital Solitary Functioning Kidney: Overview and Recommendations. Eur Urol Open Sci 25:11–20. https://doi.org/10.1016/j.euros.2021.01.003
72. Kasap-Demir B, Soyaltın E, Arslansoyu-Çamlar S, Alparslan C, et al Cardiovascular risk assessment in children and adolescents with congenital solitary kidneys. J Clin Hypertens (Greenwich). 2021 Feb;23(2):245-252. doi: 10.1111/jch.14159. Epub 2021 Jan 2. PMID: 33387392; PMCID: PMC8030084.
